# The Happy-Productive Worker Model and Beyond: Patterns of Wellbeing and Performance at Work

**DOI:** 10.3390/ijerph16030479

**Published:** 2019-02-06

**Authors:** José M. Peiró, Malgorzata W. Kozusznik, Isabel Rodríguez-Molina, Núria Tordera

**Affiliations:** 1 IDOCAL (Institut d’Investigació en Psicologia del RRHH, del Desenvolupament Organitzacional i de la Qualitat de Vida Laboral), Universitat de València & IVIE, Avda. Blasco Ibáñez 21, 46010 Valencia, Spain; 2 Research Group for Work, Organizational and Personnel Psychology (WOPP), Katholieke Universiteit Leuven, Dekenstraat 2, 3000 Leuven, Belgium; gosia.kozusznik@kuleuven.be; 3 IDOCAL (Institut d’Investigació en Psicologia del RRHH, del Desenvolupament Organitzacional i de la Qualitat de Vida Laboral), Universitat de València, Avda. Blasco Ibáñez 21, 46010 Valencia, Spain; isabel.rodriguez@uv.es (I.R.-M.); nuria.tordera@uv.es (N.T.)

**Keywords:** occupational wellbeing, performance, happy-productive worker

## Abstract

According to the happy-productive worker thesis (HPWT), “happy” workers perform better than “less happy” ones. This study aimed to explore the different patterns of relationships between performance and wellbeing, synergistic (i.e., unhappy-unproductive and happy-productive) and antagonistic (i.e., happy-unproductive and unhappy-productive), taking into account different operationalizations of wellbeing (i.e., hedonic vs. eudaimonic) and performance (i.e., self-rated vs. supervisors’ ratings). It also explored different demographic variables as antecedents of these patterns. We applied two-step cluster analysis to the data of 1647 employees. The results indicate four different patterns—happy-productive, unhappy-unproductive, happy-unproductive, and unhappy-productive—when performance is self-assessed, and three when it is assessed by supervisors. On average, over half of the respondents are unhappy-productive or happy-unproductive. We used multidimensional logistic regression to explain cluster membership based on demographic covariates. This study addresses the limitations of the HPWT by including both the hedonic and eudaimonic aspects of wellbeing and considering different dimensions and sources of evaluation. The “antagonistic” patterns identify employees with profiles not explicitly considered by the HPWT.

## 1. Introduction

Wellbeing at work can be conceptualized from two distinct perspectives based on different philosophical traditions: the hedonic view of pleasure and experience of positive affect [1] and the eudaimonic view of wellbeing as personal growth and a sense of meaning [2]. Therefore, wellbeing can be understood as having both pleasurable (or hedonic) and meaningful (or eudaimonic) components [3,4,5,6]. However, the majority of the research has studied wellbeing from the hedonic perspective, conceptualizing wellbeing as judgments and evaluations of satisfaction with some of life’s facets (e.g., job satisfaction).

According to the happy-productive worker thesis, “happy” workers should have better performance than “less happy” ones [7,8], and the quality of task performance can be influenced by the coexisting affective states [9]. This thesis has produced a series of studies [10,11] and meta-analytic research, often providing ambiguous and inconclusive results [8,12].

On the one hand, some research shows that wellbeing can predict performance. For example, studies show that when people are more satisfied with their jobs, they show higher performance [13,14]. In addition, higher positive affect has been shown to predict performance quality [15]. Furthermore, when people are more satisfied with their jobs, they show higher productivity [16] over time. People who feel better than usual at work have been found to make more effort on their tasks [17,18] and achieve a higher level of task performance [19]. In this direction, feeling active and enthusiastic in the morning has been shown to increase levels of creativity during the day [20]. Finally, positive affect has been shown to predict performance quality [21]. All these results support the HPWT, which posits that workers with higher levels of wellbeing also tend to show better performance at work, compared to workers with lower levels of wellbeing. 

On the other hand, empirical studies and meta-analyses have found the relationships between performance and job satisfaction to be spurious [22] or weak [23]. Some scholars view the connections between happiness and job performance as questionable [7], suggesting an apparently low and non-significant satisfaction–performance relationship [24]. This can be reflected by the fact that most studies that consider job satisfaction and job performance treat them as separate variables that are not directly related to each other [24]. For example, Greenberger, Strasser, Cummings, and Dunham [25] studied the causal relationship between personal control and job satisfaction, and between personal control and job performance, but they did not assume or investigate the relationship between job satisfaction and job performance [24]. There is a need to address this ambiguity in the research, and for this reason we consider it necessary to revisit and expand the happy-productive worker thesis. 

### Some Limitations of the Happy-Productive Worker Thesis

The ambiguity in the studies on the HPWT can be explained in part by the limitations of these studies [26]. First, they focus on hedonic constructs of wellbeing at the expense of eudaimonic wellbeing. In fact, most of the research has studied wellbeing from the hedonic perspective, understanding it as global evaluations of satisfaction (e.g., job satisfaction). More recently, valuable studies have revisited the thesis of the happy and productive worker, studying the possibility of expanding it conceptually to include affect [7] or alternative relationships between satisfaction and performance [8] by evaluating affective wellbeing, both as a state and a trait [19]. However, this thesis has not been extended to consider key wellbeing constructs, such as its eudaimonic dimension, which involves purpose and personal growth. Wellbeing has also been conceptualized as an eudaimonic experience of meaning at work and purpose in life [27]. This conceptualization of subjective wellbeing can be reflected in the recent progress in its measures [28], which distinguish between activities that people consider ‘pleasurable’ as opposed to the ‘worthwhileness’ or meaning at work associated with these activities [2,29,30]. Although few studies have investigated the relationship between eudaimonic wellbeing and performance [31], some research suggests that this relationship exists. For example, Niessen et al. [32] demonstrated that, on days when employees had increased perceived meaning at work, they reported being more focused on tasks and behaving in a more exploratory way, compared to days when they evaluated their work as less meaningful to them. 

A second limitation is that, in the study of the relationship between happiness and productivity, little attention is paid to a precise operationalization of productivity, and even its operationalization as job performance is far from systemic and comprehensive in terms of its dimensions or facets (e.g., in-role performance, extra-role performance, creative performance). Job performance can be understood as “a function of a person’s behavior and the extent to which that behavior helps an organization to reach its goals” [33] (p. 187). However, there is considerable debate about what work performance is. Koopmans and colleagues [34], in their systematic review, observe that, according to different studies on work performance, it can be conceptualized using the following broad dimensions: task performance, contextual performance, and counter-productive behavior. Task or in-role performance is intrinsically related to the activities included in the job description. Contextual performance refers to behaviors that are not directly related to the activities included in the job description. Organizational citizenship overlaps with the definitions of contextual performance and refers to helping others at work in the social and psychological context, thus promoting task performance [35]. Counterproductive work behaviors include behaviors such as absenteeism, theft, and substance abuse. Furthermore, creativity [36] and innovation [37] have been pointed out as another important aspect of job performance. Several authors suggest conceptualizing job performance using a broader theoretical framework, in order to mitigate error sources and find relationships between performance and job satisfaction [38]. In the present study, we incorporate different aspects of performance (in-role, organizational citizenship, and creative performance) in a global measure. Performance evaluations may come from different sources (e.g., self-assessed, supervisor, peers, customers, etc.). It is necessary to complement the employees’ self-rated performance assessment with the supervisors’ evaluation of their performance in order to avoid employee leniency or self-deception in self-ratings, which has been shown to be particularly prominent in overall or general performance assessments [39]. By including supervisors’ evaluations of their employees’ performance levels, we make sure that we are using evaluations that have been shown to have the highest mean reliability, as found in a meta-analysis by Conway and Huffcutt [40]. Therefore, the present study, in addition to employees’ self-ratings of their own performance, includes information about their performance from their direct supervisors. 

A third limitation lies in the fact that most organizational research has studied “happiness” as an antecedent of “productivity”, and only a few studies have looked for the inverse relationship [24,31]. However, there is evidence suggesting that work performance can explain wellbeing indicators. For example, evidence shows that self-rated performance predicts an increase in dedication and a decrease in emotional exhaustion over time [41]. Moreover, performance [42,43] and the experience of making progress toward one’s goals at work [44,45,46] have been shown to predict positive affective states. Additionally, studies have shown that personal initiative is positively related to an increase in work engagement over time [47]. Along the same lines, there is evidence that on days when employees were strongly focused on tasks at work, they also exhibited more vitality and learning than on days when they were weakly focused on their tasks [32]. 

A fourth limitation is that the studies from both the happy-productive and productive-happy approaches have assumed positive linear relationships, although other patterns of relations may exist, especially those that establish negative relationships between these two variables. These complex and alternative relations between these constructs require taking into consideration different configurations or patterns of these relationships, instead of analyzing them sequentially. In fact, the studies carried out within the happy productive thesis emphasize the results that confirm this thesis. These studies tend to especially explore the synergistic side of the model that produces a win-win situation for employers and employees (happy and productive), while disregarding the antagonistic or win-lose relations (happy and unproductive or unhappy and productive). However, some studies suggest that we should pay more attention to these antagonistic relations, showing, for instance, that difficulty in remembering information and poor task performance can be considered negative consequences of being “happy” at work [48]. Furthermore, other authors provide evidence of the benefits of negative affect on creative performance [49]. Based on this research, Peiró et al. [26] proposed the need to attend to not only the synergetic relations between performance and wellbeing, but also to the antagonistic ones, thus extending the propositions of the HWPW. They proposed the coexistence of four patterns of relationships between performance and wellbeing: “happy-productive”, “unhappy-unproductive”, “happy-unproductive”, and “unhappy-productive”. In fact, Ayala et al. [50] found support for these different types of patterns when considering job satisfaction and innovative performance in young employees. Moreover, they found that almost 15% of a sample of Spanish young employees fell in the group of unhappy-productive (about 9%) or the group of happy-unproductive (more than 5%). Acknowledging that the correlations between happiness and productivity are moderated, it is important to focus on the different groups of workers according to their profiles. In order “to learn more about individuals who are outside the hypothesized pattern…, it is now desirable to investigate additional measures of wellbeing and performance and identify situational and personal features associated with membership in each cluster” [51] (p. 12). 

In order to overcome the limitations of the research mentioned above, in the present study, we address them by revisiting the happy-productive worker, incorporating both the hedonic and eudaimonic components of wellbeing and considering different aspects of job performance as well as different evaluation sources. In addition, in this study, we consider wellbeing and performance simultaneously, instead of analyzing the sequence between these two constructs. To this end, we study patterns of wellbeing and performance that serve to integrate these two constructs, taking into account different operationalizations where neither of them is an antecedent of the other, in order to identify different patterns of employees, both synergistic (i.e., happy-productive) and antagonistic (i.e., unhappy-unproductive, happy-unproductive and unhappy-productive). In this way, we aim to further advance our knowledge in the direction pointed out by Warr and Nielsen [51] when they proposed identifying situational and personal features associated with membership in each cluster. More specifically, we formulate the following research questions:

- Research Question 1: Do employees show different patterns of relationships between performance and wellbeing, synergistic (i.e., unhappy-unproductive and happy-productive) and antagonistic (i.e., happy-unproductive and unhappy-productive), taking into account different operationalizations of wellbeing (i.e., hedonic vs. eudaimonic) and performance (i.e., self-rated vs. supervisor ratings)?
- Research Question 2: Will the employees remain in the same profile of wellbeing and performance in their different operationalizations?
- Research Question 3: Are there any demographic variables that may play a role as antecedents of the profiles in the different operationalizations of the “happy-productive” worker?

## 2. Materials and Methods 

### 2.1. Sample and Procedure

The members of the research team contacted several organizations, inviting them to participate in the project. Convenience sampling was used, focusing mainly on the services and production sector. The first contact was made with the general manager or the director of human resources. In a first meeting, the project, the objectives, the time required, and the procedure were explained to them. Then, if they agreed, all the workers in the organizations were invited to participate by completing the questionnaire voluntarily and confidentially.

The sample was composed of 1647 employees (52% women, 43% men, 5% information not available) from the services (81%) and production/construction (19%) sectors, working in 239 work units in different Spanish companies. With regard to age, 26% percent of participants were under 35 years old, 55% were between 35 and 50, and 16% were over 50 years old. The majority of the sample had a university degree (46%) and high school or professional training (37%). The majority were technicians/administrative workers (46%) and highly qualified professionals (24%). In addition, 62% were permanent workers, and 30% were temporary workers. The majority of the employees had worked for more than 5 years in their current position (53%). Members of the research team informed the participants on the purpose of the study, the guarantee of confidentiality and the willfulness of their participation. Participants expressed their consent to participate. The research protocol was approved by the Ethics Committee of the University of Valencia.

In this study, we used two types of informants to assess employee performance. First, we asked the employees to self-evaluate their performance. These ratings were obtained for all the employees. Second, we asked employees’ direct supervisors to rate the performance of their subordinates. In this case, performance evaluated by the direct supervisor was only obtained for 915 employees. Confidentiality of the data was guaranteed.

### 2.2. Measures

Hedonic wellbeing. Hedonic wellbeing was conceptualized as the employee’s job satisfaction, and it was measured by a 10-item reduced version of the Job Satisfaction Scale (IJSS) by Cooper, Rout and Faragher [52], referring to intrinsic job satisfaction and extrinsic job satisfaction, and one additional item measuring general job satisfaction. The score for hedonic wellbeing was the global mean score for the three types of job satisfaction. It includes items such as “Opportunity to use your skills”. The items have a seven-point Likert response format, ranging from 1 (quite dissatisfied) to 7 (very satisfied). Cronbach’s alpha for the global score of Hedonic Wellbeing was 0.87. 

Eudaimonic wellbeing. Eudaimonic wellbeing was conceptualized as a feeling of meaning and purpose at work, and it was measured by an 8-item reduced version of the scale constructed by Ryff [53], with two subscales: purpose at work and personal growth. The score for eudaimonic wellbeing was obtained by computing the global mean score for the two dimensions of the scale. It includes items such as “For me, life has been a continuous process of learning, changing, and growth”. The items have a seven-point Likert response format, ranging from 1 (strongly disagree) to 7 (strongly agree). Cronbach’s alpha for the global score of eudaimonic wellbeing was 0.72. 

Performance—rated by the employee. Employees’ self-rated work performance was operationalized as in-role performance (carrying out tasks required by the job), extra-role performance (carrying out tasks that are not required in the job description, e.g., helping others), and creative performance (carrying out tasks that are both creative and useful at work). In-role performance was measured by 3 items from a scale constructed by Williams and Anderson [54], extra-role performance was measured by 3 items from a scale by Mackenzie and colleagues [55], and creative performance was measured by a 3-item method constructed by Oldham and Cummings [36]. The composite score for performance was obtained by calculating the global mean score for the in-role, extra-role, and creative performance scales. It includes items such as: “I adequately complete assigned duties” (in-role performance); “I do not hesitate to challenge the opinions of others who I feel are leading the store/company in the wrong direction” (extra-role performance); and “How original and practical am I in my work?” The items have a seven-point Likert response format, ranging from 1 (strongly disagree) to 7 (strongly agree). Cronbach’s alpha for the global work performance score was 0.71. 

Performance—rated by the supervisor. Employee work performance evaluated by the supervisor was also operationalized as a general measure of performance quality. We measured these three aspects using three items: “What is his/her performance like?”; “What is the quality of his/her work?”; and “What was his/her level of goal achievement in the past year?” The items have a five-point Likert response format, ranging from 1 (very bad) to 5 (very good). Cronbach’s alpha for the global work Performance score was 0.89.

Demographic variables included. Organization’s sector: dummy variable (0 service, 1 production/construction). Gender: dummy variable (0 female, 1 male). Age: under 35 years old, between 35 and 50, and over 50 years old. The highest educational level achieved: no education or compulsory education, professional training or high school, advanced university degree. Occupational category: unqualified manual work, technician or administrative work, highly qualified professional, manager. Type of contract: dummy variable (0 = temporary, 1 = permanent). Seniority in the position: dummy variable (0 = less than 5 years, 1 = more than 5 years). 

### 2.3. Statistical Analysis

The sample was divided into clusters using the two–step cluster analysis method developed by Chiu and colleagues [56] in SPSS v.22 (IBM Corp., Armonk, NY, USA). The SPSS two-step cluster method is a scalable cluster analysis algorithm designed to handle large datasets, such as those analyzed in the present study. The algorithm is based on a two–stage approach: in the first stage, it undertakes a similar procedure to the k-means algorithm. In the second step, based on these results, a modified hierarchical agglomerative clustering procedure is carried out that combines the objects sequentially to form homogenous clusters [57].

The two-step clustering algorithm output offers fit information, such as the Bayesian Information Criterion (BIC), as well as information about the importance of each variable for the construction of a specific cluster [57], which is an additional attractive feature of the two-step cluster method in comparison with traditional clustering methods. Empirical results indicate that the two-step clustering method shows a near-perfect ability to detect known subgroups and correctly classify individuals into these subgroups [58]. Based on these analyses, the sample was classified into groups reflecting different configurations of wellbeing and performance dimensions. 

After finding cluster solutions for each of the combinations of variables of interest, we applied multidimensional logistic regression to explain cluster membership based on the demographic covariates described. Multinomial logistic regression is a statistical technique that specifies the dependent variable as a categorical variable that can take more than two values (in our case, the number of clusters). In multinomial logistic regression, one of the responses is chosen to serve as reference. Switching the reference group allowed us to compare the effects on all the groups. The independent variables are also categorical, with K categories. They are introduced in the model coded as k-1 binary variables. When the variables have two categories, they have been introduced as a dummy variable with a value of 0 or 1. In this case, the exponential beta coefficient represents the change in the odds of the dependent variable, associated with a one-unit change in the corresponding independent variable. When the variables have more than two categories, the coding system used is deviation coding. In this case, because there is no clear reference category, the reference category is coded as −1. This coding system compares the mean of the dependent variable for a given level to the mean of the dependent variable for the other levels of the variable. The exponential beta coefficient estimates the magnitude at which the probability of the occurrence of the event varies, comparing that category to the average of all the subjects in the study. Because the analysis does not show results for the reference group, we have repeated the analysis using the coding system with a different group as reference. With this system, we can obtain the coefficients for all the categories, which are presented in the results tables.

## 3. Results

### 3.1. Descriptive Analysis

The descriptive results are shown in Table 1 and Table 2.

### 3.2. Cluster Analyses: Different Operationalizations of the Wellbeing-Performance Patterns. 

As mentioned above, we used cluster analysis to find different patterns of relationships between performance and wellbeing, taking into account different operationalizations of wellbeing (i.e., hedonic vs. eudaimonic) and performance (i.e., self-rated vs. supervisor ratings). The results are shown below. Models 1 and 2 consider self-rated performance by the employee (hedonic wellbeing in Model 1 and eudaimonic wellbeing in Model 2). Models 3 and 4 consider performance evaluated by the supervisor (hedonic wellbeing in Model 3 and eudaimonic wellbeing in Model 4). 

When performance is evaluated by the employee, there are four clusters: (1) employees who are high in both wellbeing and high performance; (2) employees who are medium low in wellbeing and medium high in performance; (3) employees who are medium high in wellbeing and medium low in performance; and (4) employees who are low in both wellbeing and performance. 

When performance is evaluated by the supervisor, there are three clusters: (1) employees who are high in both wellbeing and performance; (2) employees who are high in wellbeing and low in performance; and (3) employees who are low both in both wellbeing and performance.

The results show that there are antagonistic patterns of wellbeing and performance (i.e., happy-unproductive, and in some cases, unhappy-productive). In fact, the results indicate that, on average, over 50% of the respondents belong to these clusters.

#### 3.2.1. Model 1: Hedonic Wellbeing vs. Self-Rated Performance (H-PE).

In Model 1, we consider two variables: hedonic wellbeing and self-rated composite performance rated by the employee. The auto-clustering algorithm indicated a four–cluster solution as the best model because it minimized the BIC value (BIC = 1060.892, BIC change from the previous cluster = −228.184). The average silhouette measure of cohesion and separation was 0.5, indicating fair to good cluster quality. The importance of both predictors was 1.00.

**Figure 1:**
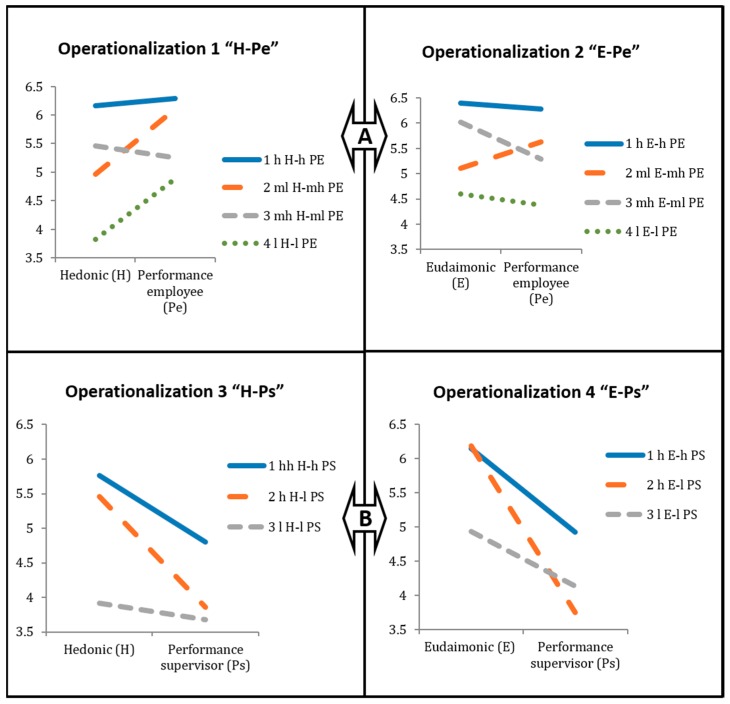
Four cluster analyses of different combinations of well-being dimensions and performance from two sources. h stands for high level; mH stands for medium high level; ml stands for medium low level; l stands for low level. H-Pe stands for Hedonic-Performance (self-rated by the Employee); E-Pe stands for Eudaimonic-Performance (self-rated by the Employee); H-Ps stands for Hedonic-Performance (evaluated by the Supervisor); E-Ps stands for Eudaimonic-Performance (evaluated by the Supervisor); A and B inside the arrows denote different types of comparisons that can be made among the different operationalizations of well-being and performance within the “happy-productive” worker framework.

Four clusters emerged (see Figure 1): (1) employees high in hedonic wellbeing (*M* = 6.17, SD *=* 0.35) and high in self-reported performance (*M* = 6.29, SD = 0.36), i.e., “hH-hPE” (*n* = 411; 24.95%); (2) employees medium low in hedonic wellbeing (*M* = 4.97, SD = 0.49) and medium high in self-reported performance (*M* = 6.10, SD = 0.31), i.e., “mlH-mhPE” (*n* = 383; 23.25%); (3) employees medium high in hedonic wellbeing (*M* = 5.45, SD = 0.46) and medium low in self-reported performance (*M* = 5.26, SD = 0.34), i.e., “mhH-mlPE” (*n* = 578; 35.09%); and (4) employees low in hedonic wellbeing (*M* = 3.82, SD = 0.71) and low in self-reported performance (*M* = 4.88, SD = 0.69), i.e., “lH-lPE” (*n* = 274; 16.67%).

#### 3.2.2. Model 2: Eudaimonic Wellbeing vs. Self-Rated Performance (E-PE).

In Model 2, we consider the following variables: eudaimonic wellbeing and self-rated performance. Although the auto-clustering algorithm indicated a two-cluster solution as the best model, we decided to opt for a four-cluster solution to maintain a similar cluster structure to Operationalization 1, and because the four-cluster solution also presented fair to good quality (BIC = 1067.114, BIC change from the previous cluster = −197.159, average silhouette measure of cohesion and separation = 0.5). The importance of both predictors was 1.00.

Four clusters emerged (see Figure 1): (1) employees high in eudaimonic wellbeing (*M* = 6.39, SD = 0.41) and high in self-reported performance (*M* = 6.27, SD = 0.34), i.e., “hE-hPE” (*n* = 596, 36%); (2) employees medium low in eudaimonic wellbeing (*M* = 5.10, SD = 0.40) and medium high in self-reported performance (*M* = 5.63, SD = 0.43), i.e., “mlE-mhPE” (*n* = 425, 26%); (3) employees medium high in eudaimonic wellbeing (*M* = 6.02, SD = 0.35) and medium low in self-reported performance (*M* = 5.28, SD = 0.36), i.e., “mhE-mlPE” (*n* = 474, 29%); and (4) employees low in eudaimonic wellbeing (*M* = 4.60, SD = 0.61) and low in self-reported performance (*M* = 4.38, SD = 0.45), i.e., “lE-lPE” (*n* = 152, 9%).

#### 3.2.3. Model 3: Hedonic Wellbeing vs. Performance Evaluated by the Supervisor (H-PS).

In Model 3, we consider two variables: hedonic wellbeing and performance assessed by the supervisor. The auto-clustering algorithm indicated a three-cluster solution as the best model because it minimized the BIC value (807.301, BIC change from the previous cluster = −172.428). The average silhouette measure of cohesion and separation was 0.5, indicating fair to good cluster quality. The importance of the predictors of hedonic wellbeing and performance evaluated by the supervisor is 1.00 and 0.91, respectively.

Three clusters emerged (see Figure 1): (1) employees high in hedonic wellbeing (*M* = 5.76, SD = 0.57) and high performance evaluated by the supervisor (*M* = 4.80, SD = 0.26), i.e., “hH-hPS” (*n* = 334, 37%); (2) employees high in hedonic wellbeing (*M* = 5.46, SD = 0.56) and low in performance evaluated by the supervisor (*M* = 3.86, SD = 0.36), i.e., “hH-lPS” (*n* = 402, 44%); and (3) employees low in hedonic wellbeing (*M* = 3.91, SD = 0.83) and low in performance evaluated by the supervisor (*M* = 3.67, SD = 0.86), i.e., “lH-lPS” (*n* = 179, 20%).

#### 3.2.4. Model 4: Eudaimonic Wellbeing vs. Performance Evaluated by the Supervisor (E-PS).

In Model 4, we consider two variables: eudaimonic wellbeing and performance evaluated by the supervisor. Although the auto-clustering algorithm indicated a four–cluster solution as the best model, we decided to opt for a three-cluster solution to maintain a similar cluster structure to operationalization 3, and because the three-cluster solution also presented fair to good quality (BIC = 786.235, BIC change from the previous cluster = −242.320, average silhouette measure of cohesion and separation = 0.5). The importance of the predictors of eudaimonic wellbeing and performance evaluated by the supervisor was 1.00 and 0.81, respectively.

The three clusters identified are (see Figure 1): (1) employees high in eudaimonic wellbeing (*M* = 6.14, SD = 0.52) and high performance evaluated by the supervisor (*M* = 4.92, SD = 0.14), i.e., “hE-hPS” (*n* = 240, 26%); (2) employees high in eudaimonic wellbeing (*M* = 6.19, SD = 0.45) and low in performance evaluated by the supervisor (*M* = 3.75, SD = 0.56), i.e., “hE-lPS” (*n* = 416, 46%); and (3) employees low in eudaimonic wellbeing (*M* = 4.93, SD = 0.52) and low in performance evaluated by the supervisor (*M* = 4.14, SD = 0.55), i.e., “lE-lPS” (*n* = 259, 28%).

### 3.3. Profiles of (un)Happy-(un)Productive Workers in Different Operationalizations of Wellbeing and Performance 

In the following section, we try to reveal on whether it is helpful to obtain different profiles of (un)happy–(un)productive workers on the basis of different operationalizations of wellbeing and performance. If the individuals remain in the same or an equivalent category regardless of the variables considered to create the groups, it would be sufficient to consider only one operationalization. In order to analyze this, we compare Models 1 and 2 (both with four clusters) and Models 3 and 4 (both with three clusters). Other comparisons do not make sense because the number of clusters is different. In fact, a different number of clusters depending on the performance measure (self-rated or evaluated by the supervisor) would mean that this operationalization is important.

In order to shed light on this issue, we present the results of the analysis of how many individuals belonging to a specific cluster in one operationalization (e.g., hH-hPE) belong to the same cluster in a different operationalization (e.g., hE-hPE), as well as how many participants belonging to one cluster in one operationalization (e.g., hH-hPS) belong to a different cluster in another operationalization (e.g., hE-lPS). The clusters found with the four types of operationalizations of the variables (dimensions of wellbeing and two sources of information about performance) can be found in the Figure 1. The results show that a large number of employees do not belong to analogous clusters in different operationalizations of wellbeing and performance. This result means that some employees are classified as both unhappy in a hedonic way and, simultaneously, happy in an eudaimonic way (and vice-versa).

#### 3.3.1. Comparison A (Model 1–Model 2): Hedonic–Employee-Rated Performance (H-PE) vs. Eudaimonic–Employee-Rated Performance (E-PE).

If the whole sample is considered, 50.6% of the respondents belong to a homologous cluster in both the H-PE and E-PE models. This means that about half of the employees had comparable wellbeing and performance profiles in both models. They have similar profiles in terms of both kinds of wellbeing. Interestingly, the other half of the employees (49.4%) do not belong to homologous clusters, which means that they belong to a cluster that suggests that they are unhappy in a hedonic way and, simultaneously, to a cluster that suggests that they are happy in an eudaimonic way, or vice versa. 

#### 3.3.2. Comparison B (Model 3–Model 4): Hedonic–Supervisor-Rated Performance (H-PS) vs. Eudaimonic–Supervisor-Rated Performance (E-PS)

Almost two thirds of the respondents (63.9%) belong to a homologous cluster in the H-PS and E-PS models, whereas 36.1% of the respondents belong to clusters with different profiles depending of the operationalization of wellbeing. This means that almost a third of the participants could be simultaneously happy in a hedonic way and unhappy in an eudaimonic way, or vice-versa, at a certain level of performance evaluated by the supervisor. 

### 3.4. Demographic Variables as Significant Antecedents of the Wellbeing—Performance Classification.

As indicated previously, we used multidimensional logistic regression to explain cluster membership based on the demographic covariates. The odds ratios for all the models are displayed in Table 3, Table 4, Table 5 and Table 6. An odds ratio greater than 1 implies that a person in a given category has greater odds of belonging to a cluster than a person in the reference category (in the case of variables with 2 categories) or than the average of all the subjects in the study (in the case of variables with more than 2 categories). An odds ratio below 1 suggests reduced odds. We identified different demographic predictors when different operationalizations of wellbeing (hedonic-eudaimonic) and performance (self- or supervisor-evaluated) are considered.

#### 3.4.1. Multidimensional Logistic Regression: Model 1 (H-PE)

The multinomial logistic regression analyses identified five predictors that explain cluster membership: the organization’s sector, gender, seniority in the position, educational level, and occupational category (see Table 3). The results show that the model has a good fit (−2 log LR = 679.06, X^2^ = 129.83, df = 24, *p* ≤ 0.001) (with LR being the likelihood ratio). The probability of having high wellbeing and high performance is greater in the production sector and for managers. The probability of having medium low wellbeing and medium high performance is greater in the production sector, for people with more than 5 years of seniority, and for technicians/administrative work. The probability of having medium high wellbeing and medium low performance is greater in the services sector, for people with less than 5 years of seniority, with professional training or high school, and for technicians/administrative workers. Finally, the probability of having low wellbeing and low performance is greater in the services sector, for men, with no education or compulsory education, and for technicians/administrative work.

Comparing Clusters 1 (high levels) and 4 (low levels), the production sector, women, people with a university degree, and managers are more likely to be in Cluster 1, whereas the services sector, men, people with no education or compulsory education, and technicians/administrative workers are more likely to be in Cluster 4.

#### 3.4.2. Multidimensional Logistic Regression: Model 2 (E-PE)

The multinomial logistic regression analyses identified five predictors that explain cluster membership: the organization’s sector, gender, age, educational level, and occupational category (see Table 4). The results show that the model has a good fit (−2 log LR = 777.45, X^2^ = 99.68, df = 27, *p* ≤ 0.001) The probability of having high wellbeing and high performance is greater in the production sector, women, and managers. The probability of having medium low wellbeing and medium high performance is greater for men, people over 50 years old, and unqualified manual workers or technicians/administrative workers. The probability of having medium high wellbeing and medium low performance is greater for women, and for unqualified manual workers or technicians/administrative workers. Finally, the probability of having low wellbeing and low performance is greater for the services sector, men, people with no education or compulsory education, and technicians/administrative workers.

Comparing Clusters 1 (high levels) and 4 (low levels), results are similar to those in Operationalization 1. The production sector, women, people with university degrees, and managers are more likely to be in Cluster 1, whereas the services sector, men, people with no education or compulsory education, and technicians/administrative workers are more likely to be in Cluster 4.

#### 3.4.3. Multidimensional Logistic Regression: Model 3 (H-PS)

The multinomial logistic regression analyses identified two predictors that explain cluster membership: type of contract and occupational category (see Table 5). The results show that the model has a good fit (−2 log LR = 68.14, X^2^ = 38.70, df = 8, *p* ≤ 0.001). The probability of having high wellbeing and high performance is greater for people with a temporary contract and for highly qualified professionals or managers. The probability of having high wellbeing and low performance is greater for people with a permanent contract and people who do unqualified manual work. Finally, the probability of having low wellbeing and low performance is greater for people with a temporary contract and for unqualified manual workers or technicians/administrative workers.

Comparing Clusters 1 (high levels) and 3 (low levels), highly qualified professionals or managers are more likely to be in Cluster 1, whereas unqualified manual workers or technicians/administrative workers are more likely to be in Cluster 3.

#### 3.4.4. Multidimensional Logistic Regression: Model 4 (E-PS)

The multinomial logistic regression analyses identified five predictors that explain cluster membership: the organization’s sector, gender, type of contract, age, and occupational category (see Table 6). The results show that the model has a good fit (−2 log LR = 358.37, X^2^ = 60.39, df = 16, *p* ≤ 0.001). The probability of having high wellbeing and high performance is greater in the production sector, women, people between 35-50 years old, people with a temporary contract, and managers. The probability of having high wellbeing and low performance is greater in the production sector, women, people under 35 years old, with a permanent contract, and who do unqualified manual work. Finally, the probability of having low wellbeing and low performance is greater for the services sector, men, people over 50 years old, with a permanent contract, and who do unqualified manual work.

Comparing Clusters 1 (high levels) and 3 (low levels), the production sector, women, people with a temporary contract, between 35–50 years old, and managers are more likely to be in Cluster 1, whereas the services sector, men, people with a permanent contract, over 50 years old, and who do unqualified manual work are more likely to be in Cluster 3.

## 4. Discussion

The aim of the present study was to revisit the happy productive worker model, extending it to consider not just the synergies between happiness and productivity, but also the antagonistic relations between these two constructs. Moreover, we aimed to clarify the implications of different operationalizations of relevant theoretically-based constructs for the model. Finally, we aimed to identify demographic antecedents for each cluster solution. In this way, this work has addressed important limitations of the happy-productive worker model by incorporating both the hedonic and eudaimonic components of wellbeing, considering different aspects of job performance as well as their different sources of evaluation, and focusing not just on the synergies between the two constructs (happiness and productivity), but also on the antagonistic relations, an issue that has hardly been considered in the research based on the model.

The results support a different way to specify and expand the happy-productive worker model. Indeed, by analyzing the relationships between different constructs, we are not taking a positive relationship that leads to being a “happy-productive” or “unhappy-unproductive” worker for granted. The present research has also contemplated a negative relationship between constructs that would appear on a daily basis and that would lead to being “happy-unproductive” or “unhappy-productive” at work. In this study, we provide an affirmative response to Research Question 1, which asks whether “employees show different patterns considering the antagonist relation beyond the traditional synergetic relation between performance and wellbeing (i.e., happy-productive)”. In fact, we have found antagonist patterns of wellbeing and performance (i.e., happy-unproductive and, in some cases, unhappy-productive) that are well represented in our sample. We found these alternative patterns by taking into account different operationalizations of wellbeing (i.e., hedonic, eudaimonic) and performance (i.e., self-rated, evaluated by the supervisor). In fact, the results indicate that, on average, over 50% of the respondents belong to the unhappy-productive/happy-unproductive clusters, which suggests that it is important to consider the antagonistic patterns of wellbeing and performance when re-defining the happy-productive worker thesis. Thus, we contribute to filling the gap identified by Warr and Nielsen [51], who pointed out that it is important to learn more about individuals who are outside the happy-productive pattern by considering additional measures of performance and wellbeing. 

In fact, Research Question 2 asks whether the same employees belong to the same patterns of wellbeing and performance in their different operationalizations. The results show that a large number of employees do not belong to analogous clusters in different operationalizations of wellbeing and performance, which means that some employees are classified as unhappy in a hedonic way and, simultaneously, happy in an eudaimonic way (and vice-versa). This result draws our attention to the complexity of the phenomenon of wellbeing and the importance of considering both the hedonic and eudaimonic dimensions in studies on wellbeing. It clearly shows that merely considering the hedonic aspect of wellbeing provides only half the picture. We believe future research should more thoroughly investigate the antecedents and outcomes for “hedonically-happy” and “eudaimonically unhappy” employees.

In addition, the results suggest that employees’ self-rated performance is often not reflected in their supervisor’s evaluation of their performance. This draws our attention to the importance of considering more than one source of evaluation of work performance in order to obtain valid information about the employees’ task performance, extra-role performance, and creativity. It is possible that the disparity in the evaluation of the employees’ performance level is due to the fact that employees might be more lenient when self-rating their general performance [38]. It is also possible that, when assessing their own performance, employees’ responses reflect not only their past behavior, but also their expectations of current and future behavior [58]. We think it would be interesting to investigate more in depth the reasons for the differences between employees’ ratings of their own performance and the ratings given by their direct supervisors. 

Finally, the results provide an affirmative response to Research Question 3 about whether there are any demographic variables that play a role as antecedents of the clusters in different operationalizations of the “happy-productive” worker. The existence of differences in the demographic variables between clusters provides yet another way to validate the clusters and the different operationalizations of wellbeing and performance. This means that it is reasonable to expand the study of employees and their different outcomes at work to different patterns of wellbeing and performance, and include alternative configurations of “happy-unproductive” and “unhappy-productive” clusters. 

Following the recommendations of Warr and Nielsen [51], we identified a number of situational and personal features associated with membership in each profile when additional measures of wellbeing and performance are considered. Our study examines whether personal features, such as gender, age, and educational level, and situational features, such as sector, type of contract, occupational category, and seniority in the position, play a predictor role in the different profiles obtained, based on the operationalizations of wellbeing (hedonic-eudaimonic) and performance (self- or supervisor- evaluated) considered. The exploratory results provide relevant information showing that occupational category is the only variable with a predictor role in the four models studied. Moreover, another situational variable (sector) and a personal variable (gender) significantly predict the profiles in three of the four models studied. Interestingly, the type of contract is a significant antecedent in the two models in which the supervisors’ performance assessment is considered, whereas the educational level is a significant antecedent in the two models where self-assessed performance is considered. More specifically, women, workers in the production sector, and management or highly qualified professionals are more likely to be included in the happy-productive profile, whereas men, workers in the services sector, employees with a low education level, and technicians/administrative workers are more likely to be included in the unhappy-unproductive cluster. 

We also identified the main features of employees included in the happy-unproductive profiles. These features differ across the four models studied. The “high hedonic/low performance (self-rated)” pattern is populated more by employees from the services sector with professional training and technician-administrative jobs. In the case of the “high eudaimonic/low performance (self-rated)” pattern, it is mostly composed of women and employees in unqualified or technician/administrative jobs. It is interesting to note that, when we look at the two similar profiles generated using supervisor ratings of performance, the employees with a higher probability of belonging to these patterns (both hedonic and eudaimonic) have permanent contracts and are employed in unqualified or manual jobs. Finally, it is interesting to identify the features that more often characterize employees included in the unhappy/productive profiles. The employees included in the “low hedonic/high performance (self-rated)” profile work in the production sector, have seniority (>5 years) and professional education, and work in technician-administrative jobs. The employees included in the “low eudaimonic/high performance (self-rated)” profile are mostly men over 50 years old working in unqualified-manual or technician-administrative jobs. Considering this complex picture of personal and situational characteristics associated with the different profiles obtained with different types of wellbeing and performance, we can conclude that the different models are not redundant, and different types of wellbeing and different sources of performance need to be considered to better understand the happy-productive worker model. Further research is needed to confirm the predictive power of the variables studied and extend the study by including other personal and situational variables, in order to better describe the employees in each profile.

In sum, the present study addresses a number of limitations of the happy productive worker thesis, and it sheds light on a number of issues that may clarify the previous inconsistencies of the model. First, this study included both the hedonic and eudaimonic aspects of wellbeing, coinciding with recent conceptualizations of wellbeing as having both pleasurable and meaningful components [3,4,5]. The identification of the hedonic “happy-productive” and “unhappy-unproductive” patterns coincides with studies indicating that there is a positive relationship between hedonic wellbeing and performance [13,14,15,16,17,18]. The identification of the “unhappy-productive” pattern agrees with research that shows a negative relationship between positive affect and the dimensions of performance [48]. Simultaneously, the identification of the eudaimonic “happy-productive” pattern supports research that suggests a synergetic relationship between eudaimonic wellbeing and performance [31]. These patterns support previous research showing that daily increases in perceived meaning at work were related to employees’ increased focus on tasks and greater exploratory behavior [31]. Second, this study considers different dimensions and sources of evaluation of employees’ performance. On the one hand, we operationalize job performance as consisting of different facets or dimensions (i.e., in-role performance, extra-role performance, creative performance) that can help to capture its manifestations. On the other hand, we consider two sources of information about performance: self-rated performance and performance rated by the direct supervisor. Third, the present research analyzes alternative configurations that have not been considered in the happy-productive worker thesis. It shows the importance of these alternative configurations, reflected by the number of employees who belong to the “happy-unproductive” and “unhappy-productive” clusters (over 50% on average), suggesting that the work reality is built on these antagonistic patterns, as well as on the synergetic ones. Thus, antagonistic patterns should not be neglected in future research. Finally, this study has identified a number of individual and situational features that significantly distinguish the different profiles in each of the operationalizations of the happy-productive worker model.

### Limitations

The current paper’s findings should be interpreted cautiously in light of several potential limitations. A limitation of the study is that most of the sample belonged to the services sector, although some of the sample is from the production sector, including areas such as construction. This limitation questions the representativeness of the results of underrepresented sectors. Services and production sectors could certainly vary in their different types of procedures and practices, such as performance evaluation or health and wellbeing promotion. The sample is more balanced in terms of gender, age, job category, or type of contract. In any case, this study represents a first approach to understanding the diversity in the patterns of relationships between performance and wellbeing in organizations. A second limitation is the fact that self-rated performance and performance rated by supervisors were not assessed with the same scale, due to the difficulties in obtaining responses from supervisors about all their subordinates (in fact, we had a high reduction in the sample when gathering data). This situation can raise some doubts about the reasons for the differences in performance-wellbeing patterns when each of the measurement methods is used. Thus, these differences could be due to different performance measures rather than to different informants. However, both measures can be considered global performance measures. Self-rated performance is a composite measure that includes the basic components of performance [34]. Performance rated by the supervisor measures global performance considering three global indicators: general performance, quality, and achievement of objectives. 

## 5. Conclusions

This study shows that the relationship between wellbeing and performance is more complex than the HPWT proposes. Different operationalizations of these constructs need to be considered. Moreover, we found that a large percentage of respondents are grouped under the happy-unproductive or the unhappy-productive profiles. The results also indicate that employees can be unhappy in a hedonic way and, simultaneously, happy in an eudaimonic way (and vice versa). Finally, we show that there are several significant antecedents of the patterns, in terms of demographic variables, in different operationalizations of wellbeing and performance. 

Future studies on the antecedents and consequences of these patterns of wellbeing and performance can be relevant for organizational practice because they might help to identify a broader scope of employees’ profiles regarding their performance and wellbeing and the circumstances in which they experience synergies and antagonisms between these two important constructs.

In conclusion, the results of this study draw our attention to the fact that there can be different typologies of “happy-productive” workers that may take into account both hedonic and eudaimonic dimensions of wellbeing, as well as two different informants about the employees’’ work performance. As we can see, a large percentage of workers do not pertain to the conventional “happy-productive” or “unhappy-unproductive” patterns, but rather to the antagonistic quadrants of “unhappy but productive” and “happy but unproductive”. 

## Figures and Tables

**Table 1:** Descriptive statistics (demographic variables).

Variables	%
Sector	
service	81
production	19
Gender	
female	52
male	43
Age	
<35 years	26
35–50 years	55
>50 years	16
Educational level	
No education or compulsory	14
Professional training or high school	37
University degree	46
Occupational category	
Unqualified manual work	10
Technician or administrative	46
Highly qualified professional	24
Manager	
Type of contract	
temporary	30
permanent	62
Seniority in the position	
<5 years	40
>5 years	53

**Table 2:** Descriptive statistics.

Feature	Mean	Standard Deviation (SD)
Hedonic wellbeing	5.25	0.91
Eudaimonic wellbeing	5.78	0.76
Performance rated by the employee	5.65	0.69
Performance rated by the supervisor	4.17	0.68

**Table 3:** Multinomial logistic regression analysis of factors associated with the clusters. Model 1: Hedonic (H) Performance employee (PE).

		(Cluster 1)			(Cluster 2)			(Cluster 3)			(Cluster 4)	
	2	3	4	1	3	4	1	2	4	1	2	3
Predictors	OR (95% CI)	OR (95% CI)	OR (95% CI)	OR (95% CI)	OR (95% CI)	OR (95% CI)	OR (95% CI)	OR (95% CI)	OR (95% CI)	OR (95% CI)	OR (95% CI)	OR (95% CI)
Sector (0 service / 1 production)		0.55 ** (0.37–0.82)	0.61 * (0.39–0.96)		0.49 *** (0.33–0.73)	0.54 ** (0.34–0.85)	1.8 ** (1.22–2.67)	2.03 *** (1.38–3.01)		1.64 * (1.04–2.58)	1.85 ** (1.17–2.91)	
Gender (0 female / 1 male)			1.61 ** (1.11–2.31)			1.44 * (1.00–2.08)			1.62 ** (1.15–2.28)	0.62 ** (0.43–0.90)	0.69 * (0.48–1.00)	0.62 ** (0.44–0.87)
Seniority (0 < 5 years / 1 > 5 years)	1.37 * (0.99–1.91)			0.73 * (0.52–1.01)	0.63 ** (0.46–0.85)			1.59 ** (1.17–2.16)	1.4 * (0.99–1.96)			0.72 * (0.51–1.01)
Educational level												
No education or compulsory			1.59 ** (1.13–2.23)			1.52 * (1.08–2.14)			1.68 ** (1.22–2.32)			
Professional training or high school					1.36 ** (1.07–1.72)			0.74 ** (0.58–0.94)				
University degree			0.61 *** (0.46–0.82)			0.54 *** (0.41–0.72)			0.67 ** (0.51–0.87)	1.63 *** (1.23–2.17)	1.83 *** (1.39–2.43)	1.5 ** (1.14–1.95)
Occupational category												
Unqualified manual work												
Technician or administrative	2.04 *** (1.55–2.69)	2.16 *** (1.67–2.80)	2.37 *** (1.69–3.32)	0.49 *** (0.37–0.65)			0.46 *** (0.36–0.60)			0.42 *** (0.30–0.59)		
Highly qualified professional												
Manager	0.53 ** (0.32–0.86)	0.51 ** (0.32–0.81)	0.33 ** (0.16–0.68)	1.89 ** (1.16–3.07)			1.96 ** (1.23–3.13)			3.07 ** (1.48–6.38)		

Reference cluster is in brackets. Cluster 1: h H- h PE; Cluster 2: ml H-mh PE; Cluster 3: mh H-ml PE; Cluster 4: l H-l PE; OR: odds ratio; CI: confidence interval; * *p* ≤ 0.05, ** *p* ≤ 0.01, *** *p* ≤ 0.001.

**Table 4:** Multinomial logistic regression analysis of factors associated with the clusters. Model 2: Eudaimonic (E) Performance employee (PE).

		(Cluster 1)			(Cluster 2)			(Cluster 3)			(Cluster 4)	
	2	3	4	1	3	4	1	2	4	1	2	3
Predictors	OR (95% CI)	OR (95% CI)	OR (95% CI)	OR (95% CI)	OR (95% CI)	OR (95% CI)	OR (95% CI)	OR (95% CI)	OR (95% CI)	OR (95% CI)	OR (95% CI)	OR (95% CI)
Sector (0 service / 1 production)	0.62 ** (0.43–0.90)	0.57 ** (0.40–0.82)	0.29 *** (0.15–0.55)	1.6 ** (1.11–2.30)		0.47 * (0.24–0.90)	1.75 ** (1.22–2.50)		0.51 * (0.26–0.99)	3.42 *** (1.81–6.46)	2.14 * (1.11–4.13)	1.96 * (1.01–3.78)
Gender (0 female / 1 male)	1.56 ** (1.16–2.10)		1.69 ** (1.11–2.58)	0.64 ** (0.48–0.86)	0.67 ** (0.49–0.91)			1.49 ** (1.10–2.03)	1.61 * (1.05–2.48)	0.59 ** (0.39–0.90)		0.62 * (0.40–0.95)
Age												
< 35 years					1.32 * (1.05–1.68)			0.75 * (0.60–0.95)				
35-50 years						1.37 * (1.02–1.84)					0.73 * (0.54–0.98)	
> 50 years	1.4 ** (1.08–1.82)			0.71 ** (0.55–0.92)	0.69 ** (0.43–0.96)	0.64 * (0.43–0.96)		1.44 *** (1.10–1.89)			1.56 * (1.04–2.35)	
Educational level												
No education or compulsory			1.7 ** (1.17–2.46)			1.84 ** (1.25–2.71)			1.82 ** (1.24–2.68)	0.59 ** (0.41–0.85)	0.54 ** (0.37–0.80)	0.55 ** (0.37–0.80)
Professional training or high school												
University degree			0.59 *** (0.43–0.81)			0.60 ** (0.43–0.84)			0.66 ** (0.47–0.92)	1.7 *** (1.23–2.35)	1.66 ** (1.19–2.33)	1.52 ** (1.09–2.12)
Occupational category												
Unqualified manual work	1.85 ** (1.24–2.76)	1.56 * (1.04–2.34)		0.54 ** (0.36–0.80)			0.64 * (0.43–0.96)					
Technician or administrative	1.3 * (1.01–1.67)	1.31 * (1.02–1.69)	1.71 ** (1.14–2.58)	0.77 * (0.60–0.98)			0.76 * (0.59–0.98)			0.58 ** (0.39–0.88)		
Highly qualified professional												
Manager	0.47 ** (0.29–0.75)	0.45 ** (0.27–0.74)	0.36 * (0.14–0.91)	2.15 ** (1.33–3.46)			2.22 ** (1.35–3.65)			2.76 * (1.10–6.91)		

Reference cluster is in brackets; Cluster 1: h E- h PE; Cluster 2: ml E-mh PE; Cluster 3: mh E-ml PE; Cluster 4: l E-l PE; OR: odds ratio; CI: confidence interval; * *p* ≤ 0.05, ** *p* ≤ 0.01, *** *p* ≤ 0.001.

**Table 5:** Multinomial logistic regression analysis of factors associated with the clusters. Model 3: Hedonic (H) Performance supervisor (PS).

	(Cluster 1)		(Cluster 2)		(Cluster 3)	
	2	3	1	3	1	2
Predictors	OR (95% CI)	OR (95% CI)	OR (95% CI)	OR (95% CI)	OR (95% CI)	OR (95% CI)
Contract (0 temporary / 1 permanent)	1.82 *** (1.26–2.62)		0.55 *** (0.38–0.79)	0.62 * (0.40–0.97)		1.6 * (1.03–2.48)
Occupational category						
Unqualified manual work	1.65 * (1.06–2.57)	2.59 *** (1.55–4.35)	0.61 * (0.39–0.94)		0.39 *** (0.23–0.65)	
Technician or administrative		1.53 * (1.04–2.25)			0.65 * (0.44–0.96)	
Highly qualified professional		0.59 * (0.38–0.93)			1.68 * (1.08–2.63)	
Manager	0.59 * (0.34–1.01)	0.42 * (0.18–0.97)	1.7 * (0.99–2.93)		2.36 * (1.03–5.41)	

**Table 6:** Multinomial logistic regression analysis of factors associated with the clusters. Model 4: Eudaimonic (E) Performance supervisor (PS).

	(Cluster 1)		(Cluster 2)		(Cluster 3)	
	2	3	1	3	1	2
Predictors	OR (95% CI)	OR (95% CI)	OR (95% CI)	OR (95% CI)	OR (95% CI)	OR (95% CI)
Sector (0 service / 1 production)		0.46 ** (0.26–0.81)		0.57 * (0.34–0.94)	2.18 ** (1.23–3.86)	1.76 * (1.07–2.92)
Gender (0 female / 1 male)		1.56 * (1.00–2.44)		1.93 *** (1.30–2.86)	0.64 * (0.41–1.00)	0.52 *** (0.35–0.77)
Contract (0 temporary / 1 permanent)	2.2 *** (1.46–3.31)	2.06 ** (1.29–3.28)	0.45 *** (0.30–0.69)		0.48 ** (0.30–0.77)	
Age						
< 35 years				0.72 * (0.54–0.96)		1.39 * (1.04–1.86)
35-50 years	0.68 ** (0.51–0.91)	0.68 * (0.50–0.93)	1.47 ** (1.10–1.95)		1.46 * (1.07–2.00)	
> 50 years		1.66 * (1.07–2.58)			0.6 * (0.39–0.94)	
Occupational category						
Unqualified manual work	1.89 * (1.11–3.20)	2.63 *** (1.50–4.63)	0.53 * (0.31–0.90)		0.38 *** (0.22–0.67)	
Technician or administrative						
Highly qualified professional						
Manager	0.55 * (0.30–0.99)	0.44 * (0.22–0.89)	1.83 * (1.01–3.34)		2.28 * (1.13–4.63)	

Reference cluster is in brackets; Cluster 1: h E- h PE; Cluster 2: h E-l PE; Cluster 3: l E-l PE; OR: odds ratio; CI: confidence interval; * *p* ≤ 0.05, ** *p* ≤ 0.01, *** *p* ≤ 0.001.
